# The Endonasal Endoscopic Management of Cerebrospinal Fluid Rhinorrhea

**DOI:** 10.7759/cureus.13457

**Published:** 2021-02-20

**Authors:** Rawan F Bubshait, Ali A Almomen

**Affiliations:** 1 Otolaryngology - Head and Neck Surgery, King Fahad Specialist Hospital, Dammam, SAU

**Keywords:** endoscopic, skull base defect, cerebrospinal fluid leak, spontaneous csf leak, traumatic csf leak, pseudotumor cerebri

## Abstract

Introduction

Cerebrospinal fluid (CSF) rhinorrhea is the result of a bony defect at the skull base with disruption of the arachnoid, dura mater, and sinonasal mucosa that leads to an active CSF leak and flow of clear fluid from the nose. The endoscopic repair of CSF leaks and skull defects have been used by an increasing number of surgeons and is the standard of care for repairing CSF leaks.

Materials and methods

We conducted a retrospective study of all cases of CSF leaks managed via the endonasal endoscopic approach from 2010 to 2020 at a tertiary referral hospital of King Fahad Specialist Hospital, Dammam (KFSH-D).

Results

Over 10 years, 61 procedures were performed on a total of 56 patients (average age, 39.9 years) with 26 spontaneous CSF leaks and 30 traumatic CSF leaks. The leak sites were frontal bone in 14% of the cases, the roof of the ethmoid in 25%, the cribriform plate of ethmoid in 39%, and the walls of sphenoid sinus in 21%; multiple site defects were found in eight patients. The defect was localized by high-resolution computed tomography (CT) of the paranasal sinuses and skull base and magnetic resonance imaging (MRI) in all patients. CT cisternography, intrathecal fluorescein injection, and topical application of fluorescein dye were used in patients as required. A combination of free grafts and flaps materials were used in most patients. A middle and inferior turbinate graft was used in 12 patients, a septal cartilage graft in 18 patients, and a pedicled nasoseptal flap in 12 patients. The success rate was 92% after the first closure attempt. A recurrence of CSF leaks was observed in four patients. The mean hospitalization time was 6.5 days. The postoperative follow-up period ranged from one year to 10 years with a mean postoperative follow-up time of three years.

Conclusions

The endonasal endoscopic approach is the current standard of care for repairing most CSF leaks and skull base defects. We have had an excellent experience with endonasal endoscopic CSF leak repair, with high success rates and low morbidity. Our results support the effectiveness and safety of this technique and should encourage otolaryngologists to apply the procedure in cases of CSF leak.

## Introduction

Cerebrospinal fluid (CSF) rhinorrhea is the result of a bony defect at the skull base with a disruption of the arachnoid, dura mater, and sinonasal mucosa that leads to an active CSF leak and flow of clear fluid from the nose. Surgical treatment of CSF leak has been historically performed through a craniotomy [1]. Dandy described the first case of intracranial repair of CSF rhinorrhea by way of a bifrontal craniotomy [2].

With the rapid development of endoscopic instrumentation, the surgical outcome has markedly improved because of better visualization of the entire sinonasal region and anatomy. The endoscopic repair of CSF leaks and skull defects has been used by an increasing number of surgeons [3-6], making it the standard of care for repairing CSF leaks.

CSF rhinorrhea occurs due to trauma in 80% to 90% of cases [7]. The most common cause is accidental trauma, followed by iatrogenic trauma, nontraumatic cases related to tumors [8-9], spontaneous cases (3% to 4%) [10], and congenital CSF leaks have also been reported. The most frequent clinical manifestation of CSF rhinorrhea is watery nasal discharge (82.7% of cases) followed by a nasal obstruction (40.7%); seizures and meningitis can also occur as the first symptoms [11]. The presence of a headache should raise the suspicion of elevated intracranial pressure (ICP) and intracranial pathology [12]. A history of a sinonasal or neurosurgical procedure, head trauma, meningitis, and intracranial or skull base tumors should raise the suspicion of CSF leak.

The aim of our research was to study and review 10 years of experience with endonasal endoscopic surgical repair of symptomatic CSF leak patients. We conducted this at a tertiary referral hospital of King Fahad Specialist Hospital, Dammam (KFSH-D), covering a population of four million in the eastern province of Saudi Arabia.

## Materials and methods

The study group consisted of patients with clinical and laboratory-confirmed CSF rhinorrhea and underwent endoscopic endonasal repair in KFSH-D between 2010 and 2020. We reviewed the files for background data, surgical technique, outcome, and postoperative course [13].

All patients included in the study underwent a careful history review, a full head and neck examination with office-based full endoscopic examination using different rigid and flexible endoscopes followed by diagnostic and localization investigations. After the endoscopic surgical intervention and successful recovery, all patients received the same postoperative management. They were prescribed bed rest for five to seven days with the head elevated 30 to 45 degrees and instructed to avoid blowing their nose, sneezing, coughing, and straining.

Stool softeners, antihistamines, and antiemetics were administered as needed for two weeks to reduce intraabdominal pressure. Antibiotics were used to prevent intracranial infection, and lumbar drain (LD) for five to seven days reduced ICP and allowed for better apposition of the graft for patients with confirmed ICP leaks [13].

The non-absorbable nasal packing was removed within three to five days. The mean duration of follow-up was two years.

## Results

Between March 2010 and January 2020, 61 endonasal endoscopic repairs of CSF leaks were performed in 56 patients (26 men and 30 women). The age of the patients ranged from four to 70 years (mean age, 39.9 years; median age, 41 years). The cause of the CSF leak was traumatic in 30 patients and spontaneous in 26 cases. Among the 30 female patients, 21 (70%) had spontaneous CSF leaks. Rhinorrhea and/or headache were the presenting symptoms in 46 (82%) cases. Ten (17%) patients suffered meningitis as a presenting symptom and complication of the disease.

The leakage site was found in the frontal bone in eight (14%) cases, in the roof of the ethmoid in 14 patients (25%), in the cribriform plate of the ethmoid in 22 patients (39%), and in the walls of the sphenoid sinus in 12 patients (21%). Eight patients had multiple site defects (Table 1).

**Table 1:** Characteristics and site of cerebrospinal fluid fistulae.

Etiology	N/Total	%
Spontaneous	26/56	46%
Traumatic	30/56	53%
Site		
Cribriform plate of the ethmoid	22/56	39%
Ethmoid roof	14/56	25%
Sphenoid sinus	12/56	21%
Frontal sinus	8/56	14%

A skull base defect was identified preoperatively by a high-resolution computed tomography (CT) scan of the paranasal sinuses and a skull base high-resolution CT and magnetic resonance imaging (MRI) in all patients. In nine patients, CT cisternography was used for accurate identification of the leakage site. Intrathecal fluorescein injection was used in five patients. In 12 patients, the leak site was identified by the topical application of fluorescein dye intraoperatively with no adverse effects.

An inferior turbinate graft was used as the graft material in six patients, middle turbinate graft in six patients, septal cartilage graft in 18 patients, pedicled nasoseptal flap in 12 patients, and a combination of free grafts and flaps materials were used in 30 patients. 

An underlay/inlay graft was used as the grafting technique in 16 patients, overlay/onlay grafts in 14 patients, and a multilayered graft and flap in 31 patients. The majority of patients had a successful endoscopic repair of the CSF leak. The CSF leak repair was successful at the first attempt in 52 patients (92%). A recurrence of CSF leaks was observed in four patients: three patients were managed at a second procedure and one patient had three endoscopic procedures due to the persistence of his increased intracranial pressure (pseudotumor cerebri) which required a permanent ventriculoperitoneal shunt.

The postoperative follow-up period ranged from one to ten years with a mean postoperative follow-up time of three years. The mean and median hospitalization times were 6.5 and four days, respectively.

## Discussion

The present study describes the 10-year experience of a tertiary referral center with endonasal endoscopic surgical closure of CSF leaks. The literature contains several algorithms for preoperative diagnosis and fistula localization.

The diagnosis of CSF rhinorrhea is both clinical and radiological. The presence of CSF in clear nasal drainage should be established by analysis for CSF markers. The most commonly used is beta-2 transferrin, for which a rapid, cost-effective test is widely available. It has been established as a sensitive and specific assay with a sensitivity of 97% and a specificity of 93% [14,15].

Another marker, the beta trace protein, can also be used for detecting a CSF leak with a slightly improved sensitivity (100%) and specificity (100%) as compared to beta-2-transferrin [15,16]. It is present in other fluids throughout the body, such as from the heart and in serum, but with a significantly lower concentration than that found in CSF [17]. 

**Figure 1:**
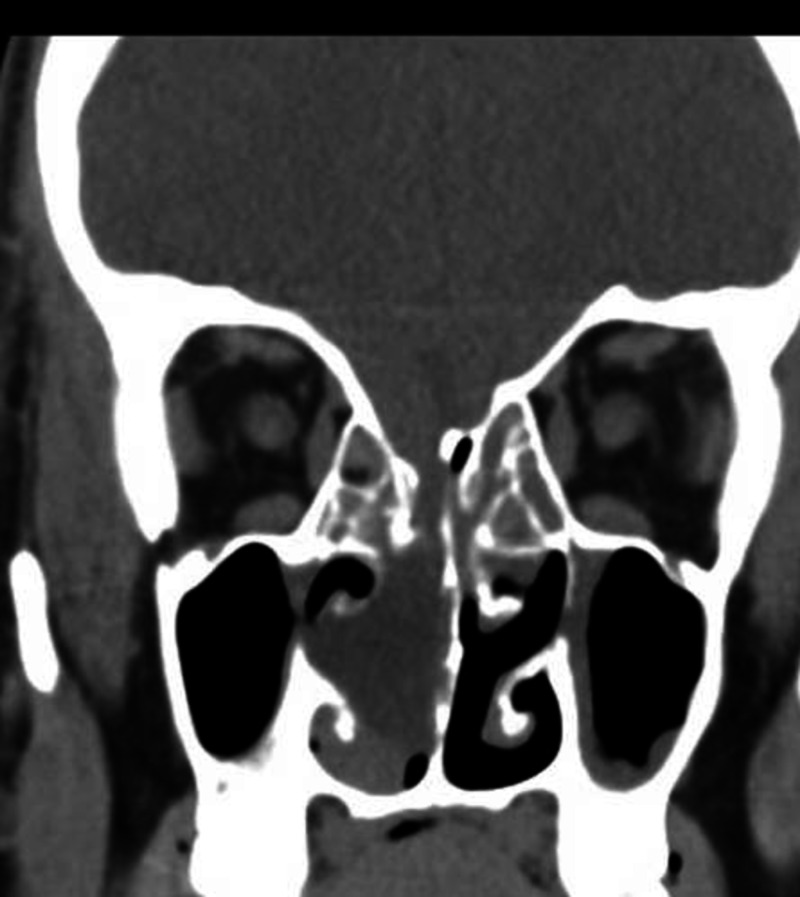
Coronal CT sinuses showing a right cribriform plate of ethmoid defect with soft tissue mass through defect. CT: computed tomography.

The localization of the leak site involves radiological investigation. As an initial procedure, numerous authors [14,18,19] have recommended a 1-mm thickness axial and coronal CT scan with a bone algorithm. A high-resolution CT scan is the imaging study of choice. It detects large bony defects, pneumocephalus, soft tissue masses, and hydrocephalus. One of the drawbacks of high-resolution CT is that it cannot firmly establish the location of a CSF leak because defects may not be actively leaking, and defects may be present without a leak (Figure 1).

**Figure 2:**
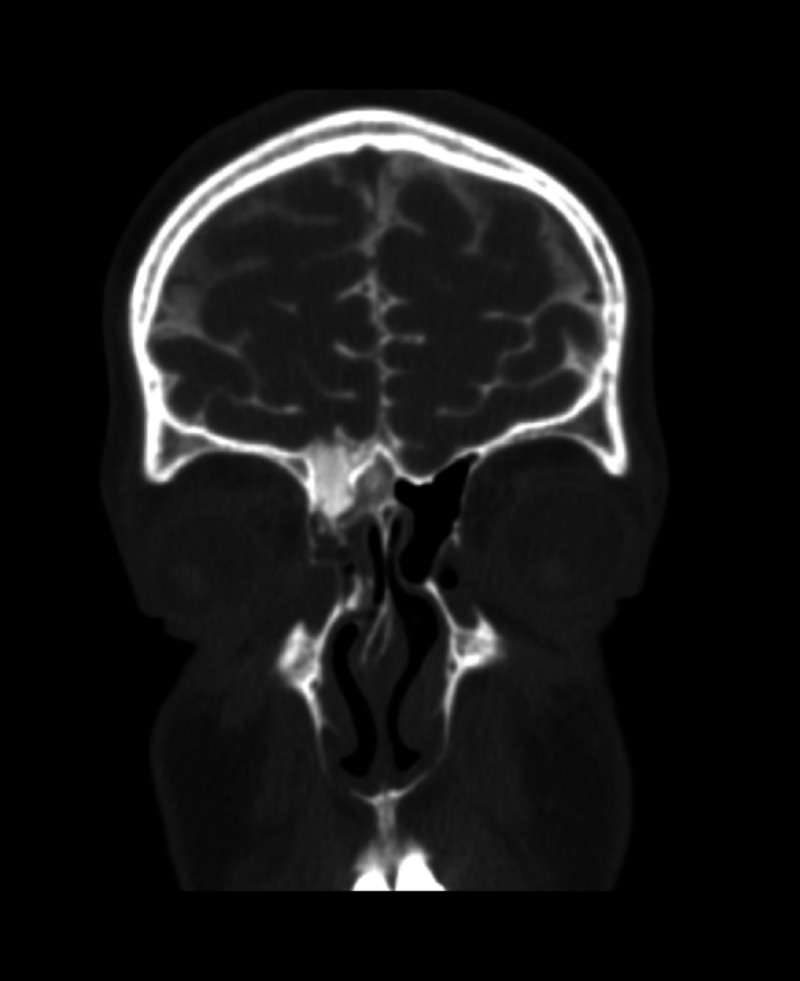
CT cisternography showing a frontal cerebrospinal fluid leak. CT: computed tomography.

CT cisternograms may provide more important information about the site of a CSF leak. However, the sensitivity of this test is quite low (48%to 96%) [20]. It is invasive and requires the injection of intrathecal contrast dye (iophendylate) prior to obtaining the CT scan (Figure 2). The contrast will be detected in the nasal cavity or a specific sinus at the completion of the study. However, the patient must be actively leaking CSF at the time of the study for it to be detected.

**Figure 3:**
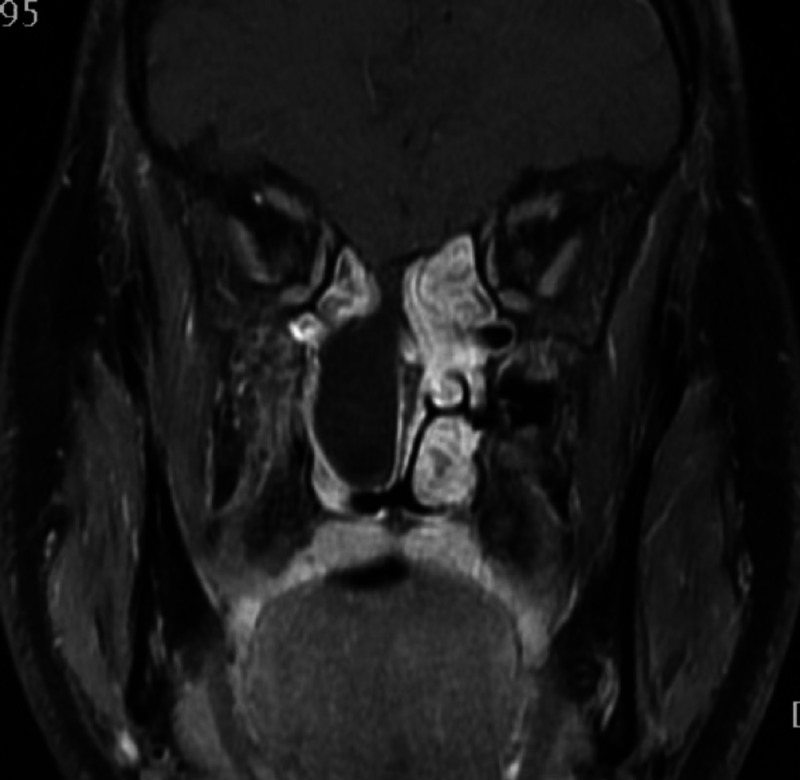
Coronal MRI sinuses confirming the presence of an ethmoid meningocele protruding through the cribriform plate defect. MRI: magnetic resonance imaging.

Additional localization techniques and the gold standard for evaluation of a CSF leak is an MRI using T2-weighted images; magnetic resonance cisternography can also help with localization (Figure 3). The sensitivity of this method is reported to be 85% to 92% with 100% specificity [21]. An MRI is effective in assessing small meningoencephaloceles and determining their composition.

Radionuclide cisternography entails the intrathecal injection of radioactive tracers (Technetium-99m). Pledgets are placed at areas suspected of a leak, and scintigrams of the skull are obtained. The pledgets are removed after 12 to 24 hours and measured for the radioactive tracer. The sensitivity is comparatively low (62% to 76%), with a 33% false-positive rate [20-23].

**Figure 4:**
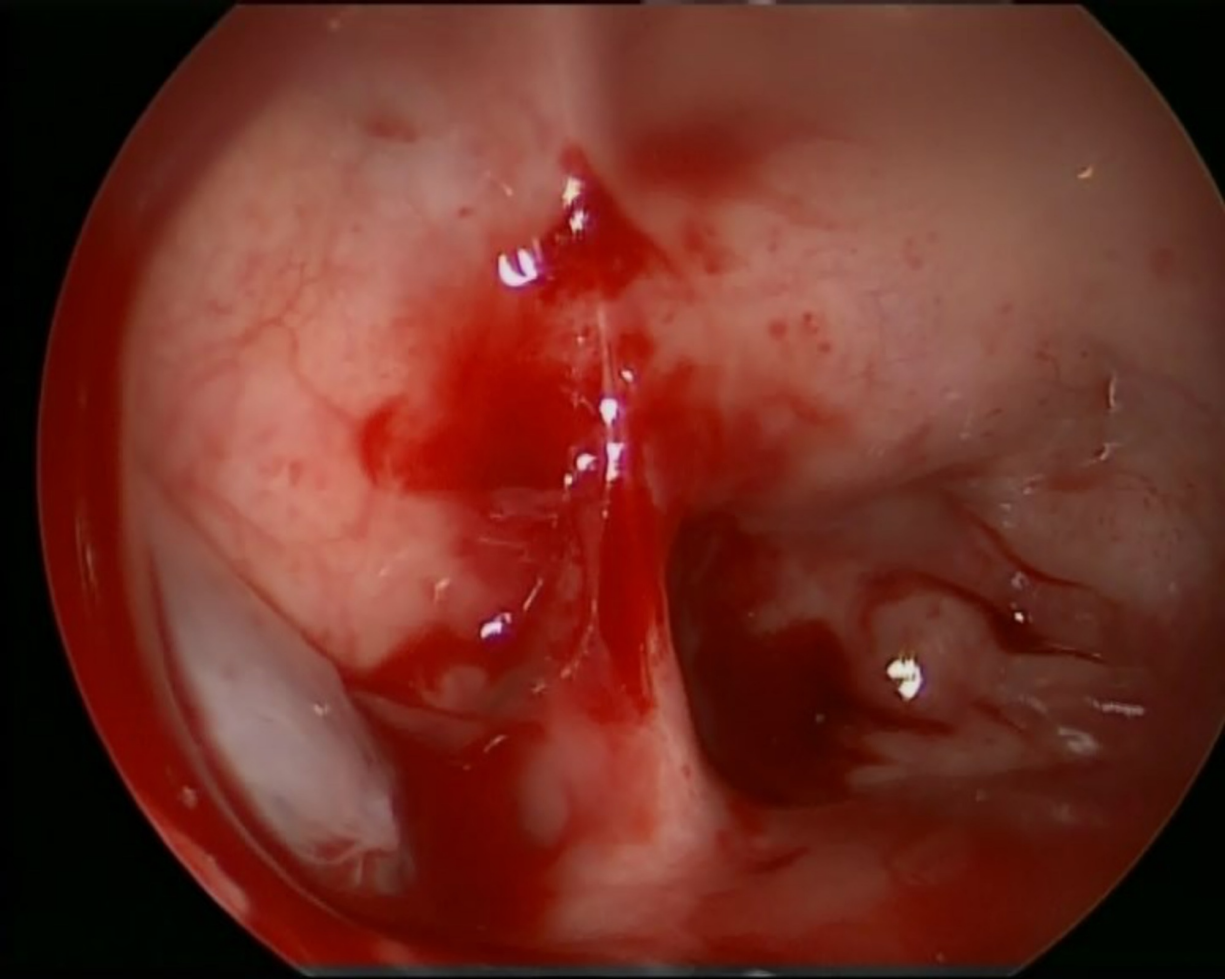
Right lateral sphenoid leak before intrathecal fluorescein dye injection.

**Figure 5:**
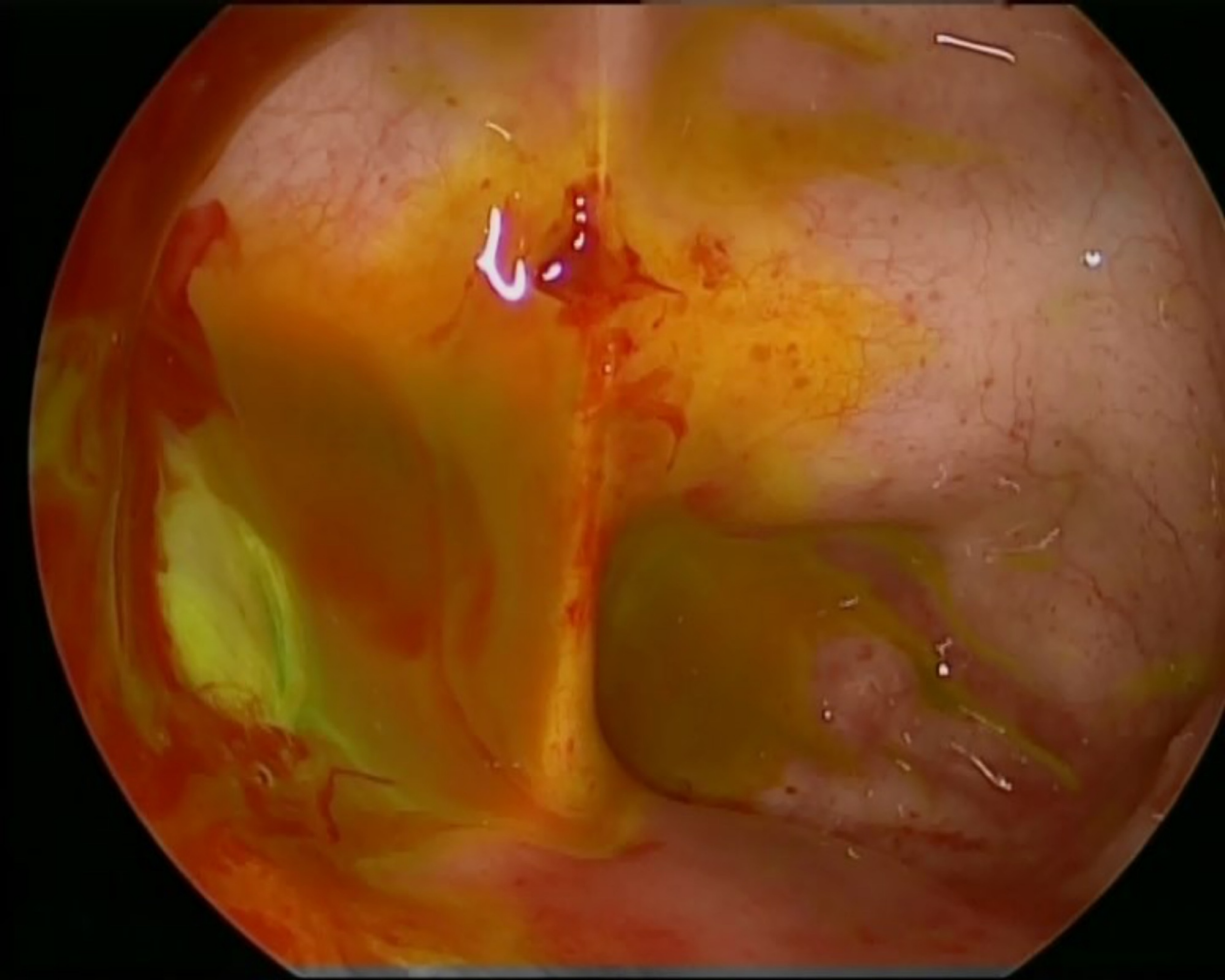
Painting of the sphenoid defect after intrathecal fluorescein dye injection.

**Figure 6:**
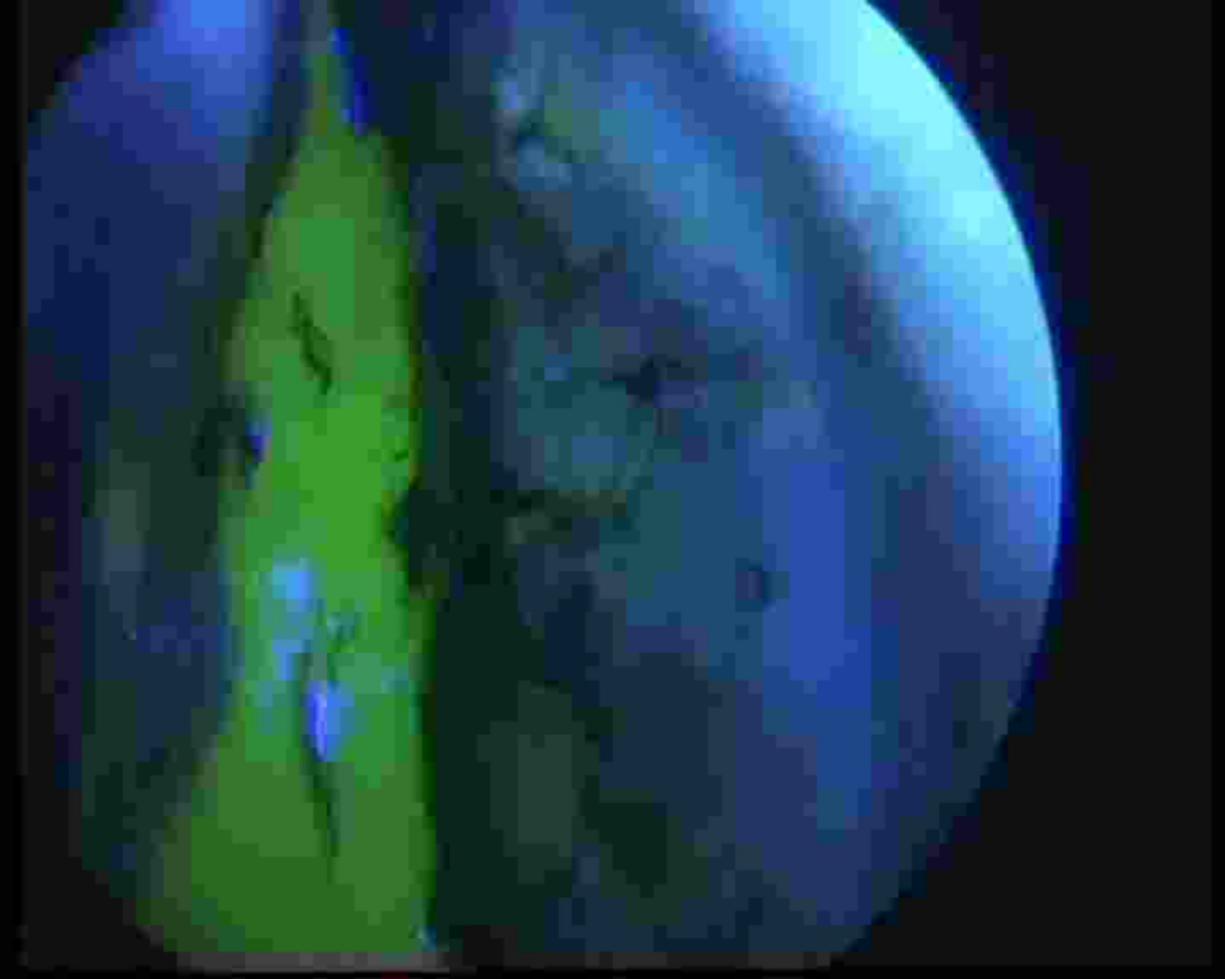
Blue light filter image with active fluorescein dye in the right cribriform plate of ethmoid cerebrospinal fluid leak.

During the intrathecal injection of fluorescein dye, CSF is withdrawn (10 ml) through an LD. This is used for mixing 0.1 ml of 10% Fluorescein dye with 10 ml of the patient’s CSF. This material is then injected intrathecally over 10 minutes and then flushed with 5 ml of the patient’s CSF. An examination using a nasal endoscope will occur 30 to 60 minutes later (Figures 4, 5). The dye can be seen without filters in most cases, but smaller defects may require a blue light filter (Figure 6).

**Figure 7:**
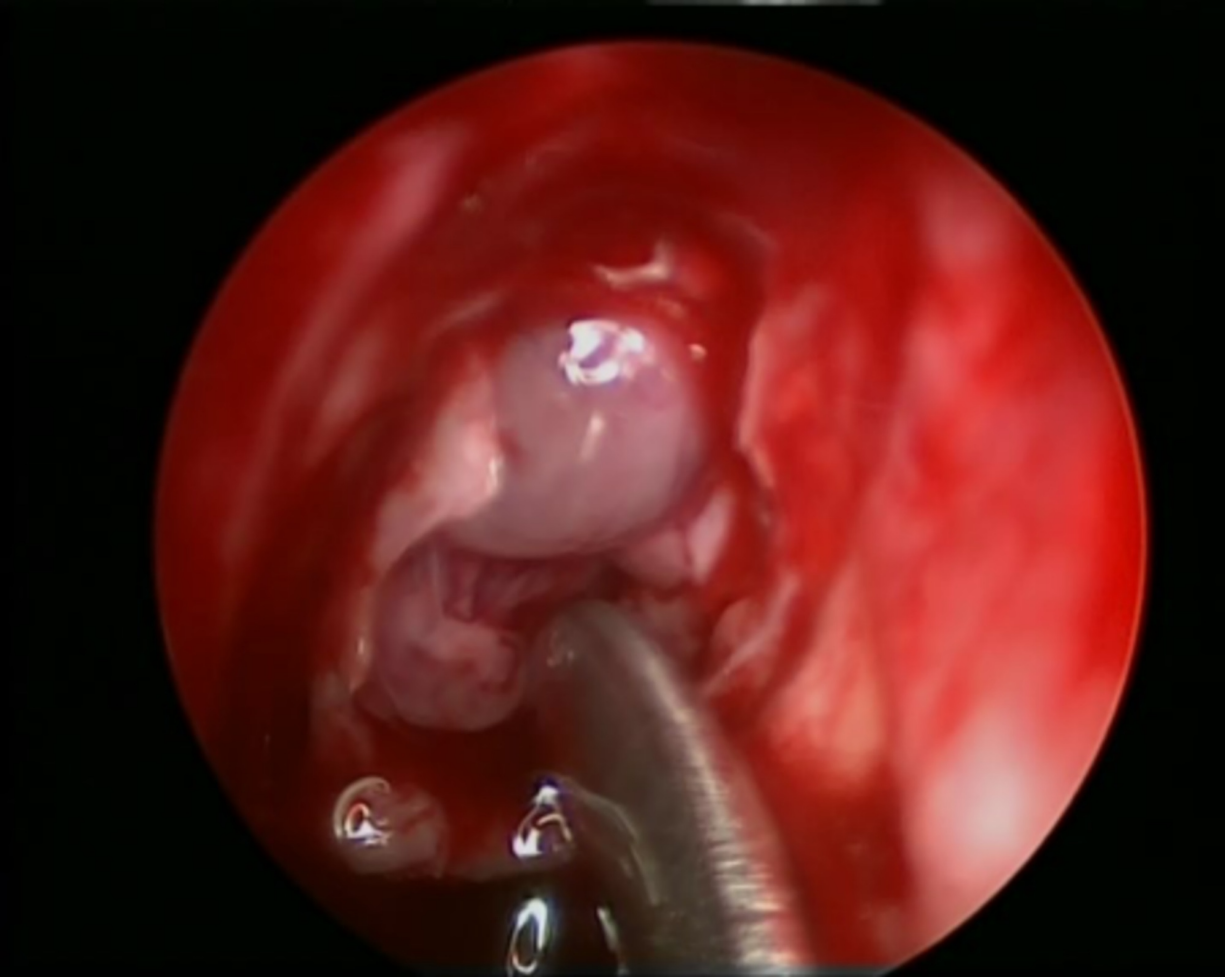
View of right ethmoid roof cerebrospinal fluid leak before the application of topical fluorescein dye.

**Figure 8:**
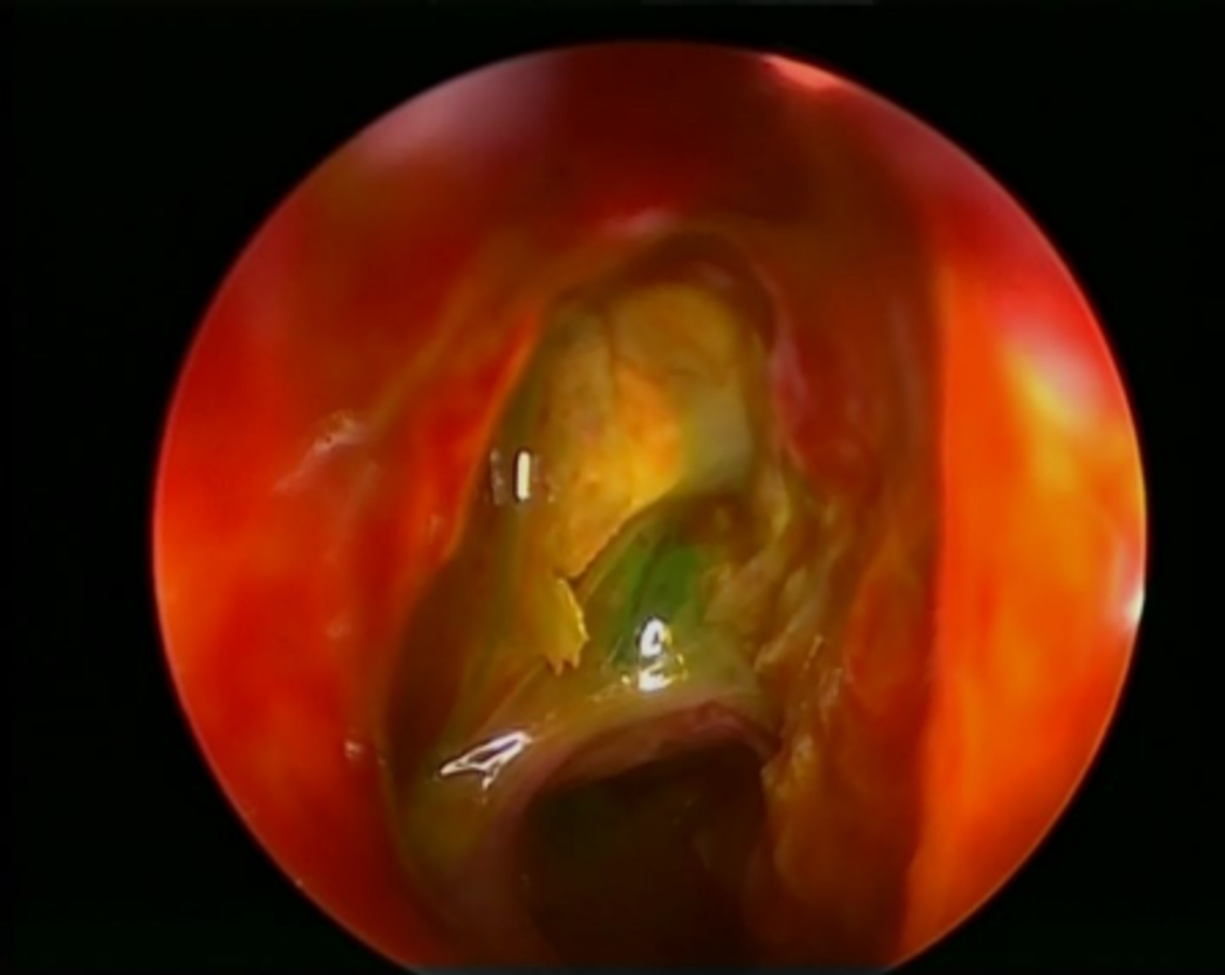
View of right ethmoid roof cerebrospinal fluid leak after the application of topical fluorescein dye.

Fluorescein is not approved by the United States Food and Drug Administration because of reported complications such as seizures, opisthotonos, and lower extremity weakness [24,25], encephalitis, and neurotoxicity with higher concentrations or rapid injections. Some authors advise using a topical application of 5% fluorescein intranasally using cotton pledgets or directly with a syringe. The change from yellow to green fluorescence indicates the presence of CSF [26] (Figures 7, 8).

We used high-resolution CT in every case, followed by MRI for better differentiation of tissue content and nature. CT cisternography was done as necessary with these methods. We successfully localized 36 leaks; intrathecal fluorescein injection was used in three patients; in 10 patients, the leak site was identified and confirmed by topical application of fluorescein dye with no adverse effects. A meticulous search identified the remaining leaks at the surgery with the help of an angled endoscope and navigation.

Conservative management of dural defects has had a success rate of only 50% [3]. It is advised for a period of four to 10 days by some authors, or for a period of two to six weeks by others. It entails bed rest and avoidance of physical exertion, prophylactic antibiotic therapy, and an LD or placement. However, it does not exclude the risk of ascending meningitis [27]. The operative management of a CSF leak is advised in the following circumstances: persistent, posttraumatic CSF leaks after four to six weeks of conservative treatment, delayed posttraumatic leaks, cases of CSF leak with a history of meningitis, all cases of spontaneous CSF fistulae, and cases with intermittent leaks [28].

Patients should be carefully selected for the endonasal endoscopic repair [13]. Contraindications to endonasal endoscopic repair include the presence of intracranial lesions, fractures of the posterior wall of the frontal sinus, lateral extensions of frontal sinus fractures, and comminuted fractures of the cranium base. Patients that have a higher tendency for surgical needs are those that are preoperatively difficult in localizing the defect or the nature of the leak site, have elevated ICP, and a large CSF leak site [13,28,29].

Numerous types of graft material have been suggested in the literature [7,14,21]. In our series, smaller defects (<0.5 cm) are managed by free onlay grafts from inferior turbinates, middle turbinate, or nasal septum. Larger defects are managed by multilayered underlay and onlay free grafts with or without pedicelled nasoseptal flaps. The versatile pedicled nasoseptal flap managed difficult-to-access and laterally placed defects like lateral pneumatized sphenoid recess leaks.

For patients suspected of elevated ICP, continuous drainage for 24 to 120 hours postoperatively is recommended by many authors in order to lower ICP and allowing for better apposition of the graft to the recipient site and attenuating sudden ICP changes [4,6,13].

Many other authors were against the routine use of an LD in all cases of skull base reconstruction [13]. Hegazy et al. [6] claimed that continuous drainage may not be necessary in every case; only 50% of their patients received continuous drainage with no decrease in postoperative success. Casiano et al. [30] reported similar results.

In our study, an LD was applied to all patients with suspected elevated intracranial pressure. In addition to the previously mentioned benefits, LD prevents side effects such as headaches, nausea, and emesis. The LD is then clamped, and the patient is carefully observed for any signs of CSF leakage in the bed rest position. Patients may slowly be elevated to a sitting position and then to a standing position as long as there is no sign of a CSF leak.

No complications were noted in any of our patients. An antibiotic was prescribed until the LD was discontinued, and packing was removed 10 to 14 days postoperatively. Some authors suggest that prophylactic antibiotics should be administered to patients with a history of meningitis and to patients who undergo recurrent closure attempts.

Patients were instructed on movement techniques to avoid breath-holding and Valsalva maneuvers. A stool softener was prescribed for every patient, and light activity was continued for six weeks after surgery. The success rates of 92% after the first attempt, rising to 97% after the second attempt, are in line with reports in the literature [4-6].

Our study was limited by its small population of 60 patients, and four were lost to follow-up and therefore excluded from the study.

## Conclusions

The endoscopic approach to the anterior skull is now considered to be the current standard of care for repairing most CSF fistulae and skull base defects. We have had an excellent experience with endonasal endoscopic CSF leak repair, with high success rates and low morbidity. Our results support the effectiveness and safety of this technique and should encourage otolaryngologists to apply the procedure in most cases of CSF leak.
